# Celastrol targets mitochondrial respiratory chain complex I to induce reactive oxygen species-dependent cytotoxicity in tumor cells

**DOI:** 10.1186/1471-2407-11-170

**Published:** 2011-05-14

**Authors:** Guozhu Chen, Xuhui Zhang, Ming Zhao, Yan Wang, Xiang Cheng, Di Wang, Yuanji Xu, Zhiyan Du, Xiaodan Yu

**Affiliations:** 1 Department of Pathology, Beijing Institute of Basic Medical Sciences, 27 Taiping Road, Beijing 100850, China

## Abstract

**Background:**

Celastrol is an active ingredient of the traditional Chinese medicinal plant *Tripterygium Wilfordii*, which exhibits significant antitumor activity in different cancer models *in vitro *and *in vivo*; however, the lack of information on the target and mechanism of action of this compound have impeded its clinical application. In this study, we sought to determine the mode of action of celastrol by focusing on the processes that mediate its anticancer activity.

**Methods:**

The downregulation of heat shock protein 90 (HSP90) client proteins, phosphorylation of c-Jun NH2-terminal kinase (JNK), and cleavage of PARP, caspase 9 and caspase 3 were detected by western blotting. The accumulation of reactive oxygen species (ROS) was analyzed by flow cytometry and fluorescence microscopy. Cell cycle progression, mitochondrial membrane potential (MMP) and apoptosis were determined by flow cytometry. Absorption spectroscopy was used to determine the activity of mitochondrial respiratory chain (MRC) complexes.

**Results:**

Celastrol induced ROS accumulation, G2-M phase blockage, apoptosis and necrosis in H1299 and HepG2 cells in a dose-dependent manner. N-acetylcysteine (NAC), an antioxidative agent, inhibited celastrol-induced ROS accumulation and cytotoxicity. JNK phosphorylation induced by celastrol was suppressed by NAC and JNK inhibitor SP600125 (SP). Moreover, SP significantly inhibited celastrol-induced loss of MMP, cleavage of PARP, caspase 9 and caspase 3, mitochondrial translocation of Bad, cytoplasmic release of cytochrome c, and cell death. However, SP did not inhibit celastrol-induced ROS accumulation. Celastrol downregulated HSP90 client proteins but did not disrupt the interaction between HSP90 and cdc37. NAC completely inhibited celastrol-induced decrease of HSP90 client proteins, catalase and thioredoxin. The activity of MRC complex I was completely inhibited in H1299 cells treated with 6 μM celastrol in the absence and presence of NAC. Moreover, the inhibition of MRC complex I activity preceded ROS accumulation in H1299 cells after celastrol treatment.

**Conclusion:**

We identified ROS as the key intermediate for celastrol-induced cytotoxicity. JNK was activated by celastrol-induced ROS accumulation and then initiated mitochondrial-mediated apoptosis. Celastrol induced the downregulation of HSP90 client proteins through ROS accumulation and facilitated ROS accumulation by inhibiting MRC complex I activity. These results identify a novel target for celastrol-induced anticancer activity and define its mode of action.

## Background

Celastrol, a quinone methide triterpene, is an active component of Tripterygium Wilfordii. Celastrol has been used in the treatment of autoimmune and neurodegenerative diseases for its antioxidative and anti-inflammatory effects [1-4]. Recently, celastrol was found to exhibit significant anticancer activities *in vitro *and *in vivo*, including the induction of apoptosis in different cancer cells [5-9], synergistically enhancing the cytotoxicity of other chemotherapeutic agents [10-12], and inhibiting the growth of glioma, melanoma and prostate cancer in nude mice [6,13,14]. However, the target and mechanism of action of celastrol are not completely clear.

Celastrol has been identified as a novel inhibitor of heat shock protein 90 (HSP90) and displays anticancer activity by inducing the degradation of HSP90 client proteins [7,9,15-17]. In addition, celastrol has been reported to be a potent inhibitor of proteasomes and induce cytotoxicity in glioma and prostate cancer models *in vivo *and *in vitro *[8,13]. Several other molecular targets have been proposed to explain the anticancer effects of celastrol, including NFκB [10,18,19], topoisomerase II [5], and xc-Cystine/Glutamate antiporter [20]. Although these targets positively correlate with celastrol-induced cytotoxicity, it is not clear which, if any, is the principal mediator of the antitumor activity of celastrol. As celastrol is moved into clinical studies, it is important to gain a better understanding of its target and mechanism.

Reactive oxygen species (ROS) are formed mainly by the interaction of oxygen molecules with electrons that escape from the mitochondrial respiratory chain (MRC) [21,22]. ROS are scavenged by antioxidative proteins, including catalase, superoxide dismutase (SOD) and thioredoxin (Trx) [23,24]. Inhibiting the activity of MRC complexes increases the leakage of electrons by blocking the electron transfer chain, thus promoting ROS production [22,25]. The downregulation of antioxidative proteins decreases ROS clearance and facilitates ROS accumulation [26]. ROS have been reported to regulate signal transduction and gene expression and to induce oxidative damage of nucleic acids, proteins, and lipids [27-29]. Therefore, ROS play an important role in the processes that determine cell fate such as proliferation, differentiation and apoptosis [30-32]. Although low levels of ROS have been reported to promote cell survival and proliferation, the accumulation of ROS induces mitochondrial-mediated apoptosis by facilitating the release of cytochrome c (Cyt c) and apoptosis-inducing factor [30,32,33].

In this study, we demonstrate that celastrol-induced cytotoxicity, including cell growth inhibition, cell cycle arrest and apoptotic and necrotic cell death, was mediated by ROS. Moreover, the downregulation of HSP90 client proteins induced by celastrol was also ROS-dependent. The accumulation of ROS leds to activation of c-Jun NH2 terminal kinase (JNK), initiation of the mitochondrial apoptotic pathway and induction of cell death. The mechanism by which celastrol induces ROS accumulation involves the inhibition of MRC complex I activity, but not the expression of antioxidative proteins. These results present a new target and mechanism for celastrol-induced cytotoxicity.

## Methods

### Cells and reagents

H1299 and HepG2 cells were obtained from the American Tissue Culture Collection (Manassas, VA). Cells were cultured with Dulbecco's modified Eagle medium (Gibco, Grand Island, NY) containing 10% fetal bovine serum (FBS, HyClone, Logan, UT). Celastrol was purchased from Calbiochem (San Diego, CA) and dissolved in DMSO. N-acetylcysteine and 17-allylamino-17-demethoxygeldanamycin were purchased from Sigma (St Louis, MO). SP600125 was purchased from LC laboratories (Worburn, MA). Z-VAD-FMK was purchased from R&D Systems (Minneapolis, MN, USA).

### Cell viability assay

Cells were collected by trypsinization and incubated with 0.4% trypan blue for 3 minutes at room temperature. The viable (unstained) cells were counted and used to calculate viability.

### Cell cycle analysis

50,000 cells were fixed with 70% ethanol containing 1% FBS at -20°C overnight, and then incubated with RNase A (20 μg/mL) at 37°C for 30 minutes, stained with propidium iodide (100 μg/mL) for 10 minutes, and then analyzed by flow cytometry (FACSCalibur, BD, USA) and ModFit LT software (FACSCalibur, BD, USA). For each measurement, 20,000 cells were analyzed and the representative measurements were shown.

### Apoptosis and necrosis analysis

Apoptosis and necrosis were determined by a Annexin V-FITC Kit (Roche, Indianapolis, IN). H1299 and HepG2 cells were collected by trypsinization. After washed with PBS, cells were stained with annexin V-FITC and propidium iodide, and then the apoptosis and necrosis were determined by flow cytometry (FACSCalibur) and CELLQuest software (FACSCalibur). For each measurement, 20,000 cells were analyzed and the representative measurements were shown.

### Measurement of mitochondrial membrane potential

The mitochondrial membrane potential was measured with a Mitochondrial Membrane Potential Assay Kit with JC-1 (Beyotime biotechnologies, Jiangsu, China) according to the manufacturer's instruction. Briefly, 50,000 cells were collected by trypsinization and incubated with JC-1 for 20 minutes at 37°C in the dark. The stained cells were washed with ice-cold working solution twice and then analyzed by flow cytometry (FACSCalibur) and CELLQuest software (FACSCalibur). 20,000 cells were analyzed in each measurement. JC-1 aggregates in the polarized mitochondrial matrix and forms J-aggregates, which emit red fluorescence at 595 nm when excited at 525 nm. However, JC-1 cannot aggregate in the depolarized mitochondrial matrix and exists as JC-1 monomers, which emit green fluorescence at 525 nm when excited at 485 nm. Mitochondrial depolarization is indicated by a decrease in the red/green fluorescence intensity ratio.

### ROS measurement

ROS were measured by a Reactive Oxygen Species Assay Kit (Applygen Technologies, Beijing, China). Briefly, cells were incubated with 3 μM DCFH-DA for 40 minutes at 37°C in the dark, and ROS were determined by fluorescence microscopy or flow cytometry (FACSCalibur) at an excitation wavelength of 480 nm and an emission wavelength of 525 nm. More than 3 fields were observed by fluorescence microscope and 20,000 stained cells were analyzed with flow cytometry in each measurement. The ROS fold was calculated based on the mean geometry fluorescence determined by flow cytometry and shown as a histogram.

### Immunoprecipitation and western blot analysis

Immunoprecipitation and western blotting were performed according to the method described by Yu [34]. For immunoprecipitation experiments, cells were lysed in TNESV buffer containing 50 mM Tris (pH 7.5), 2 mM EDTA, 100 mM NaCl, 2% Nonidet P-40 (NP-40), 1 mM Na3VO4 and cocktail (1 tablet/10 mL solution, Roche, Indianapolis, USA) at 4°C for 30 minutes. Cell lysates were centrifuged at 10,000 g to remove cellular debris. Protein concentration in the lysate was quantified with a Bicinchoninic Acid (BCA) Protein Assay Kit (Pierce, Rockford, IL, USA). Approximately 500 μg total proteins was incubated with 2 μg heat shock protein 90 antibody (Stressgen, Victoria, British Columbia, Canada) at 4°C for 12 h, after which 20 μL of protein A/G Plus Agarose (Santa Cruz, CA, USA) was added into the mixture and incubated for 2 h at 4°C. Agarose-antibody-protein complexes were washed one time with lysis buffer and two times with PBS. The immune complexes were resuspended in 20 μL of Laemmli Buffer (Bio-Rad Laboratories, CA, USA) and boiled for 5 minutes. The samples were analyzed by western blotting.

For western blotting experiments, cells were lysed in Laemmli Buffer (Bio-Rad Laboratories) and protein concentration in the lysate was quantified by a BCA Protein Assay Kit (Pierce,). Fifty micrograms of total protein was subjected to SDS-PAGE and transferred onto a PVDF membrane. The membrane was blotted with primary antibodies for 12-15 h at 4°C and incubated with horseradish peroxidase-conjugated secondary antibody for 1 h at room temperature. Proteins were detected using a SuperEnhanced Chemiluminescence Detection Kit (Applygen Technologies). The antibodies used in the study were anti-PARP, cleaved caspase 3, caspase 9, phospho-JNK, JNK, AKT, epidermal growth factor receptor, heat shock protein 90, CDK4 and Thioredoxin (Cell Signaling, Beverly, MA); anti-catalase (Abcam, Cambridge, MA); anti-Bad (Transduction Laboratories, KY); anti-cytochrome c, superoxide dismutase and Cdc37 (Santa Cruz,) and anti-β-actin (Oncogene, Boston).

### Mitochondria isolation

The mitochondria were isolated with a Mitochondrial Isolation Kit (Applygen Technologies). Fifty million cells were resuspended with ice-cold Mito-Cyto isolation buffer and homogenized with the grinder. Homogenate was centrifuged at 800 g for 10 minutes at 4°C. The supernatants were collected in a new tube, and centrifuged at 10,000 g for 10 minutes at 4°C. The supernatant and pellet were saved as cytosolic fraction and intact mitochondria, respectively. The intact mitochondria were lysed with Laemmli Buffer (Bio-Rad Laboratories) to extract mitochondrial protein. The alteration of Bad and cytochrome c in mitochondria and cytoplasm were analyzed by western blotting.

### Measurement of MRC Complexes Activity

The activity of MRC complexes was determined with Mitochondrial Respiratory Chain Complexes Activity Assay Kits (Genmed Scientifics, Shanghai, China). Briefly, the isolated mitochondria were resuspended with the Mito-Cito buffer (Applygen Technologies), frozen at -70°C and thawed at 37°C three times to extract the mitochondrial proteins. The protein concentration in the lysate was determined using the BCA Protein Assay Kit (Pierce, Rockford) and diluted to 0.1 μg/μL. The absorbance was determined on a Smartspec™ Plus spectrophotometer (Bio-Rad). The activity of complex I-linked NADH-ubiquinone reductase was determined by measuring the reduction of ubiquinone to ubiquinol, which leads to a decrease in absorbance of NADH at 340 nm. The activity was measured with or without rotenone, a specific inhibitor of NADH-ubiquinone reductase. The specific activity of complex I was calculated by subtracting the rotenone-nonsensitive activity from the total activity and is expressed as μM NADH mg/min. Complex II-linked succinate-ubiquinone reductase activity was determined by measuring the reduction of 2,6-dichlorophenolindophenol (DCIP), which can be monitored at 600 nm. The activity is expressed as μM DCIP mg/min. Complex III-linked ubiquinol cytochrome c reductase activity was determined by monitoring the reduction of cytochrome c by the electrons donated from ubiquinol, which can be monitored at 550 nm. The activity was measured with or without antimycin A, a specific inhibitor of ubiquinol cytochrome c reductase. The specific activity of complex III was calculated by subtracting the antimycin A-nonsensitive activity from the total activity and is expressed as μM CoQH2 mg/min. Complex IV-linked cytochrome c oxidoreductase activity was determined by measuring the oxidation of cytochrome c, which can be monitored at 550 nm. The activity was expressed as μM Cyt c mg/min. All measurements were performed in triplicate.

### Statistical Analysis

Statistical significance was analyzed by ANOVA test or unpaired ***t ***test. Statistical significance was defined as p < 0.01. All experiments were repeated at least three times, and data are expressed as the mean ± SD (standard deviation) from a representative experiment.

## Results

### Celastrol initiates ROS accumulation and mediates cytotoxicity in a dose-dependent manner

**Figure 1:**
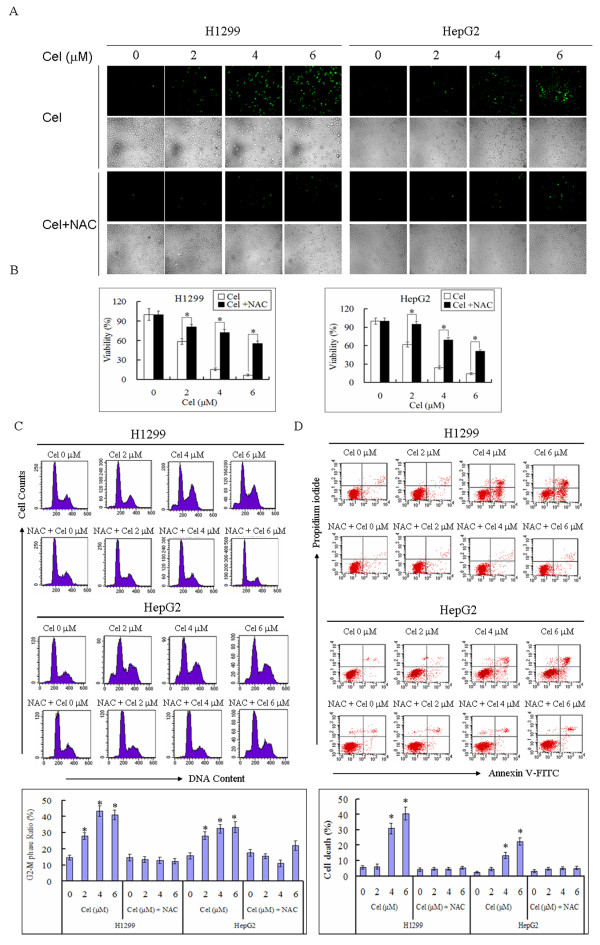
**ROS mediate cell death and growth arrest induced by celastrol**. H1299 and HepG2 cells were treated with the indicated concentration of celastrol (Cel) in the absence or presence of 5 mM NAC for 24 h. **A**. Celastrol induces dose-dependent ROS accumulation. Cells were stained with DCFH-DA and washed with PBS. More than three fields in each group were observed by fluorescence microscope (200×), and representative images are shown. **B**. ROS mediate the reduction of cell viability induced by celastrol. Viable cells were counted by trypan blue exclusion. Representative measurements of at least three independent experiments are shown. The values reported represent the mean ± SD of three separate experiments. *P < 0.01. **C**. ROS mediate G2-M phase blockage induced by celastrol. Cells were stained with PI and analyzed by flow cytometry. Representative measurements of at least three independent experiments are shown. The values reported represent the mean ± SD of three separate experiments. *P < 0.01 compared with control cells. **D**. ROS mediate apoptosis and necrosis induced by celastrol. Cells were stained with PI and annexin V-FITC and analyzed by flow cytometry. Representative measurements of at least three independent experiments are shown. The values of cell death (apoptosis and necrosis) reported represent the mean ± SD of three separate experiments. *P < 0.01 compared with control cells.

To determine the role of ROS in mediating celastrol-induced cytotoxicity, we first measured ROS levels in H1299 and HepG2 cells after celastrol exposure. As shown in Figure 1A, celastrol increased ROS levels in a dose-dependent manner in both H1299 and HepG2 cells. Celastrol also reduced cell viability in both H1299 and HepG2 cells in a dose-dependent manner (Figure 1B). Celastrol arrested cell cycle in both cell lines, and the G2-M phase ratio rose from 15 ± 1.6% to 41 ± 3.1% in H1299 cells and 15 ± 1.8% to 34 ± 3.5% in HepG2 cells after 24 h of treatment with 6 μM celastrol (Figure 1C). The results of annexin V-FITC and propidium iodide (PI) staining showed that celastrol induced apoptotic and necrotic cell death in a dose-dependent manner, and the percent of cell death (apoptosis and necrosis) was 41 ± 4.1% in H1299 cells and 22 ± 2.5% in HepG2 cells treated with 6 μM celastrol for 24 h (Figure 1D). Therefore, ROS levels positively correlate with cell death and growth arrest induced by celastrol. Moreover, scavenging of ROS by the antioxidative agent N-acetylcysteine (NAC) inhibited celastrol-induced decrease in cell viability, cell cycle arrest and cell death (Figure 1). As ROS have been reported to mediate the cytotoxicity induced by some cytotoxic agents [22,25], we assumed that ROS played a critical role in mediating celastrol-induced cytotoxicity as well.

### Celastrol-induced decrease of HSP90 client proteins is mediated by ROS

**Figure 2:**
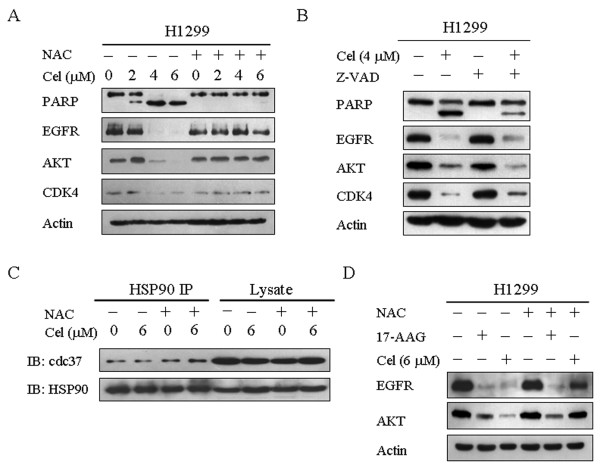
**ROS mediate the decrease of HSP90 client proteins induced by celastrol**. **A**. ROS mediate the decrease of HSP90 client proteins induced by celastrol. H1299 and HepG2 cells were treated with the indicated concentrations of celastrol in the absence or presence of 5 mM NAC for 24 h. The alteration of PARP, EGFR, AKT, CDK4 and actin was analyzed by western blotting. **B**. Celastrol-induced decrease of HSP90 client proteins is independent of apoptosis. H1299 cells were treated with or without 4 μM celastrol in the absence or presence of 50 μM Z-VAD for 24 h. The alteration of PARP, EGFR, Akt, CDK4 and actin was analyzed by western blotting. **C**. Celastrol does not disrupt the interaction between HSP90 and Cdc37 in H1299 cells. H1299 cells were treated with or without 6 μM celastrol for 12 h in the absence and presence of 5 mM NAC. Cell lysate was immunoprecipitated with an HSP90 antibody. Western blotting was used to detect HSP90 and Cdc37. **D**. NAC does not inhibit the degradation of EGFR and AKT induced by 17-AAG. H1299 cells were treated with or without 5 μM 17-AAG and 6 μM celastrol for 24 h in the absence and presence of 5 mM NAC. The alteration of EGFR, AKT and actin was analyzed by western blotting. All of the above experiments were repeated multiple times, and similar results were obtained each time; therefore, representative images are shown.

Celastrol has been identified as a novel HSP90 inhibitor and mediates cytotoxicity by facilitating the degradation of HSP90 client proteins [7,9,15-17]. Thus, we determined whether ROS were involved in mediating the decline of HSP90 client proteins induced by celastrol. Levels of the HSP90 client proteins including AKT, epidermal growth factor receptor (EGFR) and CDK4 were all decreased following celastrol treatment in H1299 cells (Figure 2A). However, the decrease of HSP90 client proteins induced by celastrol was completely inhibited by NAC (Figure 2A). To determine whether the decline of HSP90 client proteins induced by celastrol was dependent on apoptosis, we analyzed the effect of celastrol on HSP90 client protein expression in the absence and presence of Z-VAD-FMK (Z-VAD), a pan-caspase inhibitor. As shown in Figure 2B, 50 μM Z-VAD significantly decreased the cleavage of PARP (apoptosis marker) induced by celastrol, indicating that Z-VAD inhibited celastrol-induced apoptosis. However, Z-VAD had no significant effect on celastrol-induced decrease of HSP90 client proteins including EGFR, AKT and CDK4, suggesting that celastrol-induced decrease of HSP90 client proteins is not dependent on apoptosis. By using a GST pull-down assay, a previous study has reported that celastrol could disrupt the HSP90/Cdc37 complex [15]; however, we did not observe that the interaction between endogenous HSP90 and Cdc37 was disrupted by celastrol in H1299 cells (Figure 2C). In addition, we compared the effect of NAC in reversing the decrease of HSP90 client proteins induced by 17-allylamino-17-demethoxygeldanamycin (17-AAG), a classic HSP90 inhibitor, with that induced by celastrol. Both 17-AAG and celastrol induced the depletion of HSP90 client proteins including AKT and EGFR whereas NAC blocked celastrol-induced depletion of ATK and EGFR but had no effect on 17-AAG-induced HSP90 client protein degradation (Figure 2D). These data indicate that celastrol-induced inhibition of HSP90 chaperone function is mediated by ROS.

### Celastrol induces ROS accumulation by inhibiting the activity of MRC complex I

**Figure 3:**
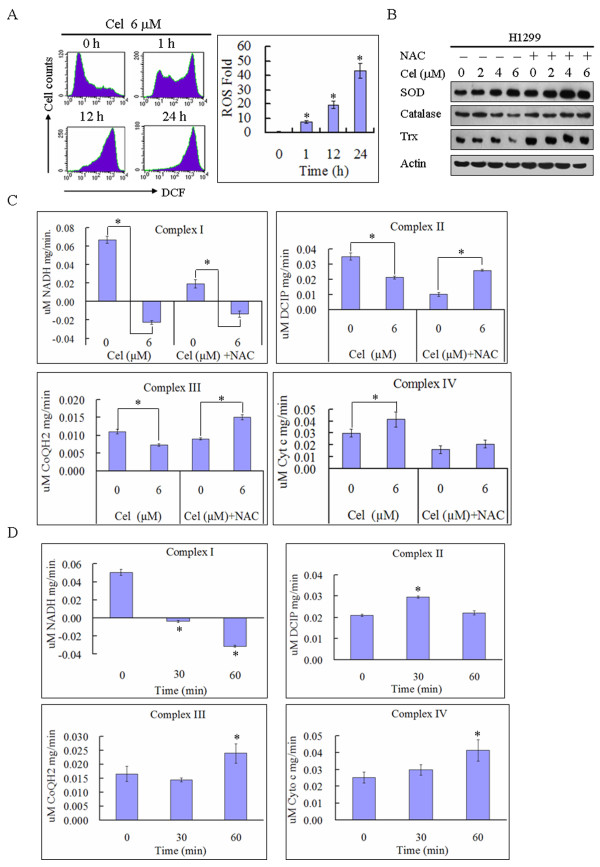
**Inhibition of MRC complex I activity is involved in mediating ROS accumulation induced by celastrol**. **A**. Celastrol induces time-dependent ROS accumulation. H1299 cells were treated with 6 μM celastrol for the designated time. Cells were stained with DCFH-DA and analyzed by flow cytometry. The relative levels of ROS geometry fluorescence are shown as a ratio compared to the control group. Representative measurements of at least three independent experiments are shown. The values reported represent the mean ± SD of three separate experiments. *P < 0.01 compared with control cells. **B**. The alteration of antioxidative proteins induced by celastrol is not involved in promoting ROS accumulation. H1299 cells were treated with the indicated concentration of celastrol in the absence or presence of 5 mM NAC for 24 h. The alteration of SOD, catalase, Trx and actin was analyzed by western blotting. Representative images of at least three independent experiments are shown. **C**. Celastrol significantly inhibits MRC complex I activity. H1299 cells were treated with or without 6 μM celastrol in the absence or presence of 5 mM NAC for 12 h. The activity of MRC complexes was measured with a Mitochondrial Respiratory Chain Complex Enzyme Activity Assay Kit and is displayed as a histogram. All columns display the mean ± SD from three independent experiments. *P < 0.01. **D**. Inhibition of MRC complex I activity is involved in mediating ROS accumulation induced by celastrol. H1299 cells were treated with 6 μM celastrol for the indicated time. The activities of MRC complexes were measured by Mitochondrial Respiratory Chain Complex Enzyme Activity Assay Kits and are displayed as a histogram. All columns display the mean ± SD from three independent experiments. *P < 0.01 compared with control cells.

ROS play a critical role in mediating the cytotoxicity induced by celastrol, but the targets by which celastrol induces ROS accumulation are unknown. To identify the targets for celastrol-induced ROS accumulation, we first measured ROS levels in H1299 cells at different time points after celastrol exposure. As shown in Figure 3A, celastrol induced time-dependent ROS accumulation in H1299 cells, and ROS levels increased 7.7 ± 0.7 times after 1 h of celastrol treatment. The downregulation of antioxidative proteins results in ROS accumulation [24]; therefore, we investigated the effect of celastrol on the expression of antioxidative proteins, including catalase, SOD, and Trx. Celastrol had no significant effect on SOD but slightly downregulated catalase and Trx expression (Figure 3B). However, the downregulation of catalase and Trx was completely inhibited by NAC (Figure 3B). Therefore, celastrol does not cause ROS accumulation by downregulating the antioxidative proteins SOD, catalase, and Trx.

Inhibiting the activity of MRC complexes promotes ROS production [25]. Therefore, we speculated that celastrol may induce ROS accumulation by inhibiting MRC complex activity. We detected the effect of celastrol on different MRC complexes. Celastrol completely inhibited MRC complex I activity but increased MRC complex IV activity (Figure 3C), indicating that complex I, but not complex IV, may be the target of celastrol in mediating ROS accumulation. The activity of MRC complex II and III decreased in H1299 cells treated with celastrol; however, NAC reversed the inhibition (Figure 3C), indicating that complex II and III are not likely targets of celastrol for ROS accumulation. In addition, MRC complex I activity was completely inhibited as early as 30 minutes after celastrol exposure (Figure 3D), which precedes ROS accumulation. In contrast, the activity of MRC complex II, III and IV did not significantly decrease after 30 or 60 minutes of treatment with celastrol (Figure 3D), further confirming that complex I is the target of celastrol in promoting ROS production.

### Celastrol-induced activation of the mitochondrial apoptotic pathway is mediated by ROS

**Figure 4:**
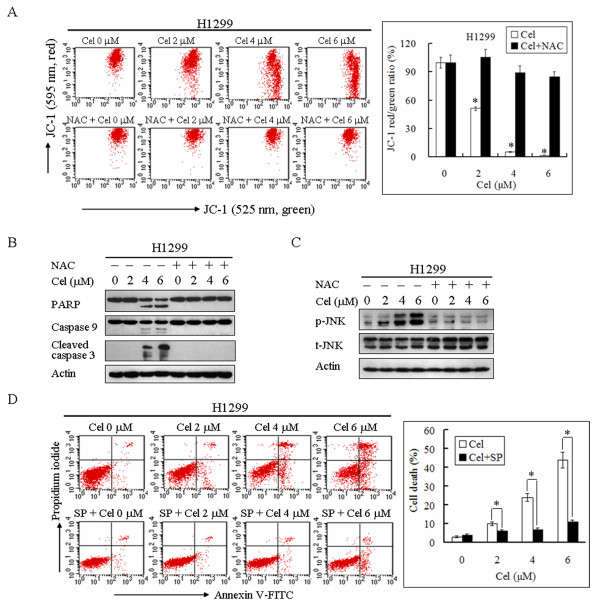
**Celastrol induces apoptosis through ROS accumulation and subsequent JNK activation**. **A**. ROS mediate the depolarization of mitochondria induced by celastrol. H1299 cells were treated with the indicated concentration of celastrol in the absence or presence of 5 mM NAC for 24 h. Cells were stained with JC-1 and analyzed by flow cytometry. The ratio of JC-1 red/green fluorescence intensity was normalized by comparing it to the control group and is represented as loss of MMP. Representative measurements of at least three independent experiments are shown. The values reported represent the mean ± SD of three separate experiments. *P < 0.01 compared with the control group. **B**. Celastrol induces caspase-dependent apoptosis. H1299 cells were treated with the indicated concentration of celastrol in the absence or presence of 5 mM NAC for 24 h. Actin and the cleavage of PARP, caspase 9 and caspase 3 were analyzed by western blotting. Representative images of at least three independent experiments are shown. **C**. ROS mediate JNK activation induced by celastrol. H1299 cells were treated with the indicated concentration of celastrol in the absence or presence of 5 mM NAC for 12 h. The alteration of phospho-JNK (p-JNK), total JNK (t-JNK) and actin were analyzed by western blotting. Representative images of at least three independent experiments are shown. **D**. JNK mediates celastrol-induced cell death. H1299 cells were treated with the indicated concentration of celastrol in the absence or presence of 40 μM SP for 24 h. Cells were stained with PI and annexin V-FITC and analyzed by flow cytometry. Representative measurements of at least three independent experiments are shown. The values reported represent the mean ± SD. *P < 0.01.

Because celastrol induced apoptosis in a dose-dependent manner (Figure 1D), we further investigated the effect of celastrol on mitochondria. The mitochondrial membrane potential (MMP) was measured using a JC-1 fluorescent probe, and the JC-1 red/green fluorescence intensity ratio was used to represent MMP. As shown in Figure 4A, celastrol treatment resulted in a dose-dependent decrease in MMP in H1299 cells. The JC-1 red/green fluorescence intensity ratio in H1299 cells treated with 6 μM celastrol for 24 h decreased to 2 ± 0.2%, indicating that celastrol significantly induced dysfunction of mitochondria. As before, NAC inhibited the decrease of MMP induced by celastrol (Figure 4A), indicating that ROS were involved in mediating mitochondrial depolarization. Moreover, as shown in Figure 4B, NAC could completely block the cleavage of PARP, caspase 9, and caspase 3 in H1299 cells treated with celastrol. These data are consistent with celastrol induced mitochondrial-mediated apoptosis through ROS accumulation.

### Celastrol activates JNK through ROS accumulation, which plays a critical role in mediating the activation of mitochondrial apoptotic pathway

As the stress-activated protein kinase, JNK can be activated by oxidative stress induced by accumulated ROS [35,36]. Activated JNK initiates the mitochondrial apoptotic pathway by mediating the activation and mitochondrial translocation of proapoptotic proteins including Bax and Bad [37-39]. We determined the effect of celastrol on JNK activation. As shown in Figure 4C, celastrol increased the level of JNK phosphorylation in a dose-dependent manner whereas NAC completely inhibited celastrol-induced JNK phosphorylation, demonstrating that JNK was activated by celastrol-induced ROS accumulation. The results of annexin V-FITC and PI staining showed that JNK inhibitor SP600125 (SP) significantly inhibited celastrol-induced apoptosis and necrosis. The percentage of cell death (apoptosis and necrosis) was 43 ± 4.1% in cells treated with celastrol and 11 ± 1.2% in cells treated with celastrol and SP (Figure 4D).

**Figure 5:**
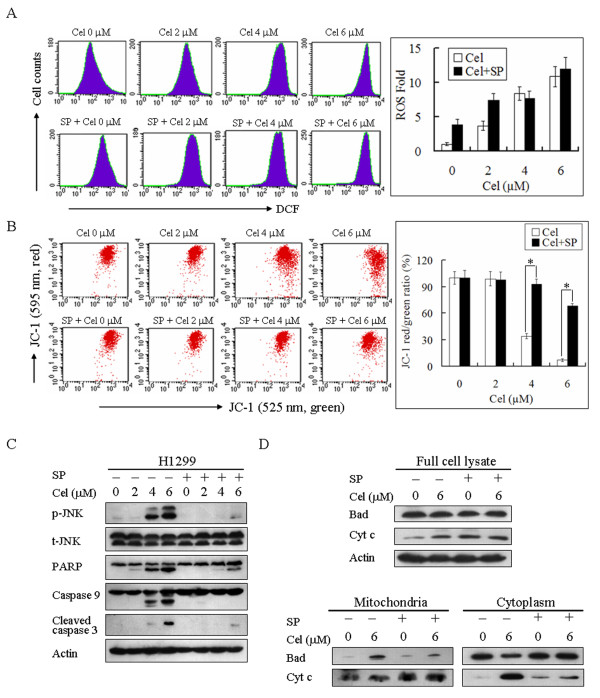
**JNK mediates celastrol-induced cell death by initiating the mitochondrial apoptotic pathway**. H1299 cells were treated with the indicated concentration of celastrol in the absence or presence of 40 μM SP for 12 h or 24 h. **A**. SP does not suppress celastrol-induced ROS accumulation. After 12 h of treatment, cells were stained with DCFH-DA and analyzed by flow cytometry. The relative levels of ROS geometry fluorescence are shown as a ratio compared to the control group. Representative measurements are shown. Each experiment was performed in triplicate, and the values reported represent the mean ± SD. **B**. JNK mediates mitochondrial depolarization induced by celastrol. Cells were stained with JC-1 and analyzed by flow cytometry. The ratio of JC-1 red/green fluorescence intensity was normalized by comparing the data to the control group and is represented as loss of MMP. Representative measurements are shown. Each experiment was performed in triplicate, and the values reported represent the mean ± SD. *P < 0.01. **C**. JNK mediates celastrol-induced apoptosis by initiating the mitochondrial apoptotic pathway. Actin, p-JNK, t-JNK and the cleavage of PARP, caspase 9 and caspase 3 were analyzed by western blotting. Representative images of three independent experiments are shown. **D**. JNK mediates mitochondrial translocation of Bad and cytoplasmic release of Cyt c induced by celastrol. Mitochondria and cytoplasm were separated and used to extract protein. The alteration and subcellular localization of Bad and Cyt c were analyzed by western blotting. Representative images of three independent experiments are shown.

We further determined the mechanism by which JNK mediates celastrol-induced cell death. As shown in Figure 5A, SP did not suppress celastrol-induced ROS accumulation, indicating that SP does not inhibit celastrol-induced cell death by suppressing ROS accumulation. However, SP significantly inhibited celastrol-induced depolarization of mitochondria. The JC-1 red/green fluorescence intensity ratio was 7 ± 1.4% and 68 ± 4.2% in H1299 cells treated with 6 μM celastrol alone and 6 μM celastrol with SP for 24 h, respectively (Figure 5B). Moreover, SP inhibited celastrol-induced JNK phosphorylation and cleavage of PARP, caspase 9 and caspase 3 (Figure 5C). Furthermore, SP significantly inhibited mitochondrial translocation of Bad and cytoplasmic release of Cytochrome c induced by celastrol (Figure 5D). Therefore, ROS-induced JNK activation played a crucial role in initiating mitochondria-mediated apoptosis of H1299 cells treated with celastrol.

## Discussion

Celastrol displays significant anti-cancer activity *in vivo *and *in vitro *in various cancer models [5,7,13,18], but the precise target and mechanism are not clear. In this study, we demonstrate that celastrol induces cytotoxicity by causing ROS accumulation. Accumulated ROS inhibited HSP90 function, activated JNK, and induced cell cycle arrest and apoptotic and necrotic cell death. The effects of celastrol on ROS accumulation and apoptosis were observed in both lung cancer cells (H1299) and hepatoma cells (HepG2). Therefore, we conclude that ROS represent a novel intermediate in mediating celastrol-induced cytotoxicity.

As the stress-activated protein kinase, JNK plays a critical role in mediating apoptotic cell death [36]. It has been previously reported that celastrol activates JNK by suppressing the transcriptional activity of ATF2 [6]. However, we observed that JNK was activated by celastrol-induced ROS accumulation. Recent studies have indicated that ROS play important roles in regulating both normal cellular processes, such as proliferation, differentiation and migration, and disease progression, such as cancer [30-33]. Accumulated ROS have been identified as the key intermediate for the cytotoxicity induced by many chemotherapeutic agents [22,38,40]. In cells, many antioxidative enzymes respond to scavenge ROS and maintain low levels of cellular ROS. Among the antioxidative system, catalase, SOD and Trx are the main proteins involved in ROS clearance, and some chemotherapeutic agents induce ROS-dependent cytotoxicity by downregulating the expression of these antioxidative proteins [23,24,41,42]. However, in our study, we found that celastrol did not affect the expression of SOD and only slightly downregulated catalase and Trx expression at a high dose, which was inhibited by NAC. Therefore, we concluded that celastrol did not induce ROS-dependent cytotoxicity by inducing dysfunction of the antioxidative system. Though many enzymes, including NADPH oxidase, cytochrome c oxidase and lipoxygenase, respond to ROS production, the main source of ROS generation is the MRC [21]. Some cytotoxic agents, such as rotenone, have been shown to induce ROS-dependent cytotoxicity by targeting MRC complex I [22,25]. In this study, we found that after exposure of 6 μM celastrol for 30 minutes, the cellular activity of MRC complex I was completely inhibited (Figure 3C). This inhibitory effect was not reversed by NAC, demonstrating that inhibition of MRC complex I activity contributes to celastrol-induced ROS accumulation. However, whether celastrol can directly inhibit MRC complex I activity requires further study.

Previous studies have reported that celastrol inhibits lipid peroxidation in rat liver mitochondrial membranes induced by ADP and Fe^2+ ^[43,44]. These results conflict with our results that celastrol induces oxidative stress by causing ROS accumulation in H1299 and HepG2 cells. It is possible that this discrepancy may be attributed to the difference between normal tissues (rat liver mitochondrial membrane) and cancer cells (non-small lung cancer cells and hepatoma cells). Celastrol has been reported to protect neuronal cells from cytotoxicity by increasing the expression of heat shock proteins and suppressing the release of inflammatory intermediates [1,3]. However, celastrol inhibits pro-inflammatory cytokine secretion and promotes expression of heat shock proteins at nanomolar concentrations whereas the cytotoxic concentration of celastrol for neuronal cells is approximately 1 μM [2,3]. Therefore, it seems that the protective or cytotoxic role of celastrol is dependent on the dose.

HSP90 is a highly abundant molecular chaperone that maintains the stability and activity of multiple kinases, transcription factors and steroid receptors [45,46]. The classic HSP90 inhibitors, such as geldanamycin (GA) and 17-AAG, inhibit HSP90 chaperone function by competing with ATP for the ATP-binding pocket of HSP90 and then facilitate the degradation of HSP90 client proteins [47]. Celastrol has been identified as an inhibitor of HSP90 and displays cytotoxicity by inducing the degradation of HSP90 client proteins [7,9,16]. Consistent with these previous reports, we found that celastrol induced the downregulation of EGFR, AKT and CDK4. However, we found that celastrol-induced decrease of HSP90 client proteins was inhibited by scavenging ROS. Recently, GA and 17-AAG have been shown to promote superoxide generation [48,49], but it has been suggested that oxidative stress alone is insufficient to disrupt HSP90 binding to its client proteins [48]. Consistent with this result, our data also show that NAC could not block the degradation of AKT and EGFR induced by 17-AAG. Compared with 17-AAG, ROS obviously play a key role in mediating the downregulation of HSP90 client proteins induced by celastrol.

Although a previous study attributed the mechanism for celastrol-induced degradation of HSP90 client proteins to the disruption of HSP90/Cdc37 complex [7], we did not find that the interaction of HSP90 and Cdc37 was disrupted by 6 μM celastrol, either in the absence or in the presence of NAC. Another report also verified that 5 μM celastrol had no effect on the interaction of HSP90 and Cdc37; only 10 μM celastrol showed the ability to decrease the interaction of HSP90 and Cdc37 [15]. Therefore, celastrol-induced disruption of the HSP90/Cdc37 complex appears to be highly dose-dependent. Because the dose required to disrupt the HSP90/Cdc37 complex is higher than the IC50 [7], it is not clear whether the cytotoxic effect of celastrol is due to the disruption of the interaction between HSP90 and Cdc37. In view of our finding that 6 μM celastrol did not disrupt the interaction between HSP90 and Cdc37, we conclude that ROS mediate the degradation of HSP90 client proteins through other pathways. It is possible that aberrant ROS cause oxidative damage to proteins, which promotes their degradation [50].

## Conclusions

**Figure 6:**
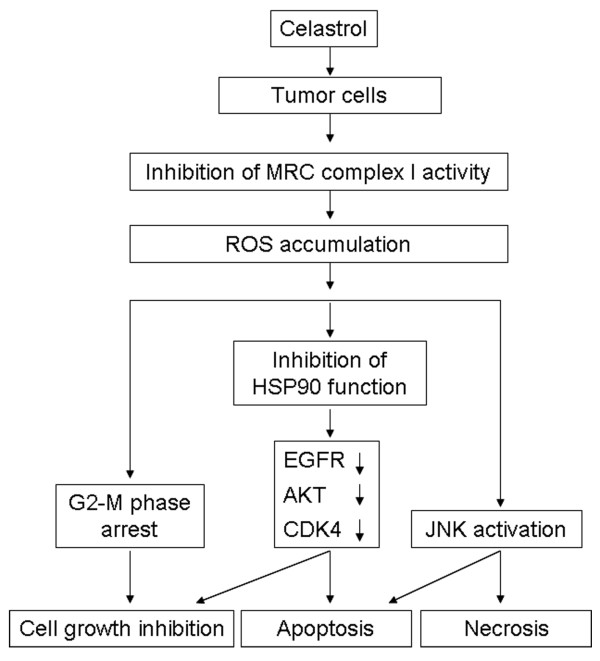
**Model of ROS-mediated cytotoxicity induced by celastrol**. Celastrol facilitates ROS accumulation in tumor cells by inhibiting the activity of MRC complex I. ROS then induce apoptosis and necrosis by activating JNK and downregulating HSP90 client proteins including EGFR, AKT and CDK4. ROS also mediate cell growth arrest by inducing G2-M phase blockage in tumor cells after celastrol treatment.

In conclusion, we demonstrate that celastrol targets MRC complex I and induces ROS accumulation and that accumulated ROS act as the key intermediate that induces cell cycle arrest, inhibits HSP90 chaperone function, downregulates HSP90 client proteins, activates JNK and initiates apoptotic and necrotic cell death (Figure 6). Therefore, the present study identifies a new target and a new mechanism for the anti-cancer activity displayed by celastrol.

## List of abbreviations

HSP90: heat shock protein 90; JNK: c-Jun NH2-terminal kinase; MMP: mitochondrial membrane potential; MRC: mitochondrial respiratory chain; NAC: N-acetylcysteine; PARP: poly (ADP-ribose) polymerase; PBS: phosphate-buffered saline; ROS: reactive oxygen species; SP: SP600125.

## Competing interests

The authors declare that they have no competing interests.

## Authors' contributions

GC designed the study, performed most of the experiments, analyzed the data and drafted the manuscript. XY participated in the design of the study, revised the manuscript and coordinated the study. XZ participated in fluorescence microscopy. MZ participated in western blotting. XC and DW participated in cell culture and drug treatment. YW participated in discussion of the data. YX and ZD participated in sample preparation. All authors read and approved the final manuscript.

## Pre-publication history

The pre-publication history for this paper can be accessed here:

http://www.biomedcentral.com/1471-2407/11/170/prepub
